# Microbial Polysaccharide-Based Formulation with Silica Nanoparticles; A New Hydrogel Nanocomposite for 3D Printing

**DOI:** 10.3390/gels9050425

**Published:** 2023-05-19

**Authors:** Maria Minodora Marin, Ioana Catalina Gifu, Gratiela Gradisteanu Pircalabioru, Madalina Albu Kaya, Rodica Roxana Constantinescu, Rebeca Leu Alexa, Bogdan Trica, Elvira Alexandrescu, Cristina Lavinia Nistor, Cristian Petcu, Raluca Ianchis

**Affiliations:** 1 Advanced Polymer Materials Group, Faculty of Applied Chemistry and Materials Science, Politehnica University of Bucharest, 1–7 Polizu Street, 01106 Bucharest, Romania; maria_minodora.marin@upb.ro; 2 Department of Collagen, National Research and Development Institute for Textile and Leather, Division Leather and Footwear Research Institute, 93 Ion Minulescu Str., 031215 Bucharest, Romania; madalina.albu@icpi.ro (M.A.K.);; 3 National Research and Development Institute for Chemistry and Petrochemistry ICECHIM—Spl. Independentei 202, 6th District, 0600021 Bucharest, Romania; 4 eBio-Hub Research Center, University Politehnica of Bucharest—CAMPUS, 6 Iuliu Maniu Boulevard, 061344 Bucharest, Romania; gratiela87@gmail.com; 5 Research Institute of University of Bucharest (ICUB), University of Bucharest, 030018 Bucharest, Romania; 6 Academy of Romanian Scientists, 010719 Bucharest, Romania

**Keywords:** salecan, silica nanoparticles, 3D printing

## Abstract

Natural polysaccharides are highly attractive biopolymers recommended for medical applications due to their low cytotoxicity and hydrophilicity. Polysaccharides and their derivatives are also suitable for additive manufacturing, a process in which various customized geometries of 3D structures/scaffolds can be achieved. Polysaccharide-based hydrogel materials are widely used in 3D hydrogel printing of tissue substitutes. In this context, our goal was to obtain printable hydrogel nanocomposites by adding silica nanoparticles to a microbial polysaccharide’s polymer network. Several amounts of silica nanoparticles were added to the biopolymer, and their effects on the morpho-structural characteristics of the resulting nanocomposite hydrogel inks and subsequent 3D printed constructs were studied. FTIR, TGA, and microscopy analysis were used to investigate the resulting crosslinked structures. Assessment of the swelling characteristics and mechanical stability of the nanocomposite materials in a wet state was also conducted. The salecan-based hydrogels displayed excellent biocompatibility and could be employed for biomedical purposes, according to the results of the MTT, LDH, and Live/Dead tests. The innovative, crosslinked, nanocomposite materials are recommended for use in regenerative medicine.

## 1. Introduction

For obtaining three-dimensional (3D) objects from a computer design, 3D printing was created more than 30 years ago. A quick and affordable design cycle is made possible by this layer-by-layer method for creating customized biomaterials [1,2]. Many polymers have been employed to create materials used in pharmaceutical and medical fields using three-dimensional printing technology [3]. Moreover, multiple materials can be combined in a single structure using multi-material 3D printing [4]. With the development of 3D printing technology, it is now possible to reconstruct tissues using active components and a growing variety of materials that have strong and stable mechanical properties, and good biocompatibility [5,6]. It is interesting how the development of functional hydrogels opens up several opportunities for incorporating hydrophilic networks into 3D-printed scaffolds that are similar to the extracellular matrix [7]. 

Hydrogels are three-dimensional polymeric structures that can absorb large amounts of water without disintegrating or losing their physical integrity [8,9]. Hydrogels have drawn a lot of interest as prospective biomaterials for medical applications such as drug delivery, tissue engineering, cell culture scaffolds, wound dressings, and filtration/separation techniques due to their high water content and inherent mechanical strength [10,11]. Hydrogels can be made from synthetic or natural polymers using techniques that include physical crosslinking, chemical gelation, or self-assembly [8,12,13,14]. Natural polymer-based hydrogels, particularly those made of polysaccharides, have seen increased use in recent years because of their outstanding biocompatibility, bioactivity, biodegradability, hydrophilicity, and low toxicity [9,15,16]. In previous research works, polysaccharides such as starch, dextran, cellulose, and their derivatives have been used to develop polysaccharide-based hydrogels [17,18]. Moreover, polysaccharides feature active groups that can be functionalized by utilizing a variety of methods in order to improve their mechanical properties and stability; furthermore, they can be produced from renewable sources [19]. Alginate, chitosan, cellulose, k-carrageenan, pectin, and other polysaccharides are frequently used in the manufacture of composite hydrogels [20,21,22,23,24]. 

Salecan is a new water-soluble glucan (natural polysaccharide), produced by the strain *Agrobacterium* sp. ZX09, which has recently been identified and commercialized [25,26,27]. Its backbone features a distinctive design “→ 3)-β-D-Glcp-(1 → 3-[β-D-Glcp-(1 → 3)-β-D-Glcp-(1 → 3)]3-α-D-Glcp-(1 → 3)-α-D-Glcp-(1→”, groups that are all connected by α-(1–3) and β-(1–3) glycosidic connections [28]. Salecan, which differs significantly from the other β-glucans, offers a variety of benefits including biocompatibility, great immune boosting, high solubility, antioxidation properties, and biodegradability. The medical, pharmaceutical, cosmetic, and food sectors all greatly value these qualities [29,30,31]. One of the most important characteristics of salecan is the rheological property, which is incredibly valuable in our current inquiry. For drug delivery purposes, our research team has developed polymer nanocomposites containing salecan [32]. We recently investigated the possibility of synthesizing exclusively salecan green crosslinked materials, as well as the first use of salecan in additive manufacturing [25].

Organic-inorganic nanocomposite particles have attracted a lot of attention recently in research areas and industry. The combined and, in some cases, synergistic effects of the organic and inorganic components in these hybrid materials make them useful for a variety of applications, including catalysis, coatings, photonics, and biomedicine [33,34]. To create organic-inorganic nanocomposite materials, several inorganic particles, including silica, Laponite clay, magnetite, zinc oxide, titanium oxide, and graphene oxide, have been used [33,35] 

The silicate minerals that make up the majority of the silica in the Earth’s crust are also present in plants and grains [36,37]. Due to the presence of silanol groups (Si-OH), silica nanoparticles (SiO_2_) have good biocompatibility, homogeneous pore size, adjustable particle size, a large surface area, and are easily modifiable [38]. Furthermore, the proliferation and differentiation of cell cultures can all be aided by the addition of silica nanoparticles [39]. Based on these features, the inorganic silica skeleton is more stable than conventional degradable biomaterials in the presence of temperature changes, organic solvents, and acidic environments [40]. The production of silica nanoparticles has increased, making them the second most common nanomaterial produced globally [41]. Functionalized silica nanoparticles, present free OH groups on their surface, and these groups are highly affine to the COO–groups found in biopolymers such as sodium alginate, gelatin, agar, etc., forming hydrogen bonds [39]. For example, Roopavath et al. prepared a 3D printable alginate/gelatin/SiO_2_ ink with increased viscosity that presented a potential for bone regeneration and nanomedicine [39]. Moreover, using a salecan graft copolymer and Fe_3_O_4_@SiO_2_ nanoparticles as the drug carrier, Hu et al. created a pH-sensitive magnetic composite hydrogel [17]. 

In the present work, a novel 3D-printed nanocomposite hydrogel was synthesized and characterized. The main objective of this study was to introduce silicon dioxide nanoparticles in the polymer network of a microbial polysaccharide (salecan) in order to obtain new 3D printable nanocomposite hydrogels. Thus, we have followed enhanced printing fidelity by varying the amount of nanofiller in the polysaccharide formulation. As far as we know, our study is the first report investigating an ink based on salecan with entrapped silica nanoparticles developed for additive manufacturing with potential for biomedical applications.

## 2. Results and Discussion

### 2.1. Rheological Behavior

All of the samples displayed normal filaments and kept the 3D-printed structure they were originally created with depending on the composition. As was seen both during and after the 3D printing process, the 3D printed shape demonstrated good stability and maintained its design. Even though they can be printed up to 10 layers thick, the neat hydrogel sample S0 and S1 showed collapsed layers in contrast to the nanocomposite structures made from the printing inks S2 and S3.

**Figure 1:**
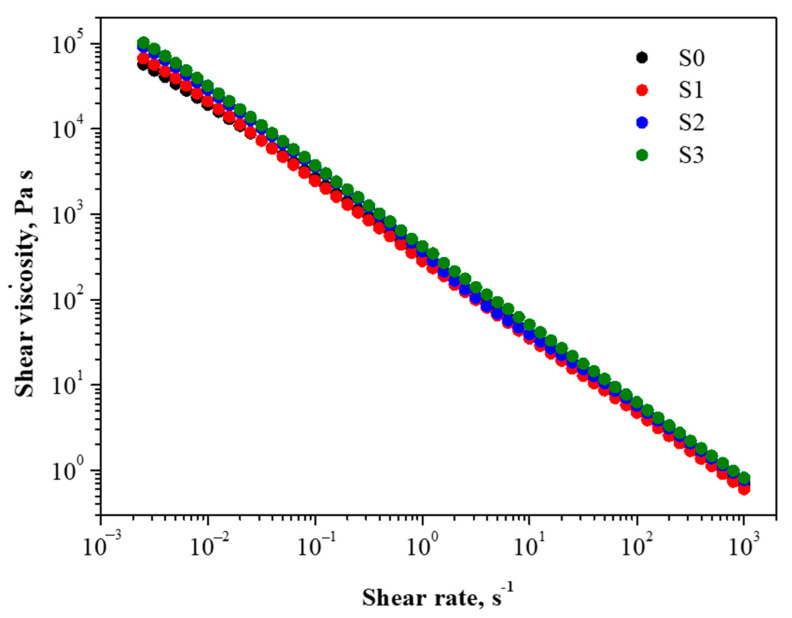
Viscosity as a function of shear rate for S0, S1, S2, and S3 hydrogel-based inks.

To evaluate the printability of the newly formulated inks and the influence of silica nanoparticles amount over the salecan, the shear viscosity was investigated. Figure 1 shows the variation of viscosity with a shear rate for S0, S1, S2, and S3. The bicomponent samples forming the printing inks offer specific rheological characteristics. The shear viscosity decreases with the applied shear rate for all samples, which suggests a non-Newtonian pseudoplastic fluid with a shear-thinning character. This rheological characteristic is typical of polysaccharide materials and is linked to their vast hydrodynamic size, which comes from the grouping of linear and stiff macromolecules, which results in high viscosities and pseudoplasticity [25,42]. Furthermore, the salecan hydrogels’ viscosity is reduced from 10^5^ Pa·s to 10^0^ Pa·s with an increased shear rate, displaying shear-thinning behavior and suggesting the feasibility of injecting salecan hydrogel-based inks [25].

As intended, the viscosity of the silica—salecan composites slightly increased with the increasing silica concentration at a constant polysaccharide concentration. The difference between the studied samples is the viscosity value, which was the highest for S3 and the lowest for S0, while S1 and S2 presented intermediate values. The slightly increased viscosity conferred by the inclusion of silica nanoparticles is beneficial for the extrusion of hydrogel and for producing 3D structures with precise geometry [43].

### 2.2. FT-IR Analyses 

**Figure 2:**
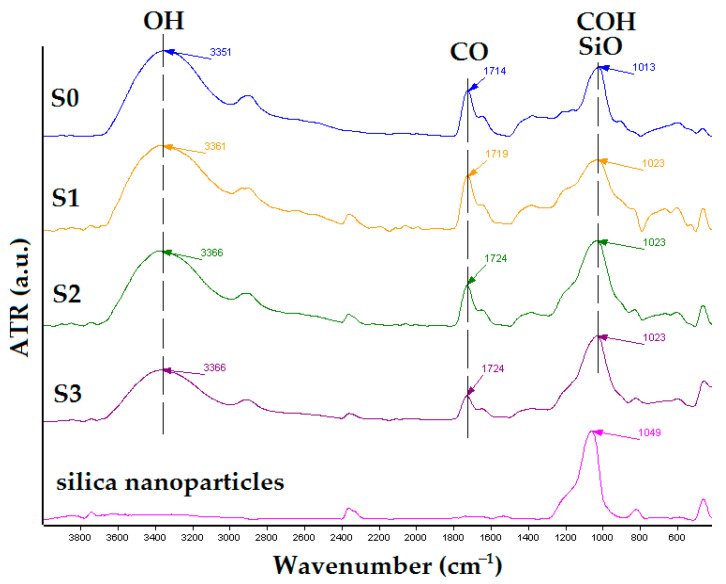
FTIR Spectra of the obtained samples.

To understand the influence of silica nanoparticle content on the structural properties of the newly obtained salecan/silica composite, we investigated the compositional behaviors of designed hydrogels by using the FT-IR technique. The FT-IR spectra of the newly obtained biomaterials based on salecan and silica nanoparticles are given in Figure 2.

FT-IR spectroscopy showed the specific peak of salecan at around 3300 cm^−1^ due to its abundant OH groups [17]. Other peaks appear at 2800 and 1013 cm^−1^, which are attributed to CH_2_ and C–OH stretching frequencies in the glucopyranose ring from the salecan structure, respectively [25,30]. Moreover, stretching vibration of the C=O peak from the citric acid structure appears on the FT-IR curve at around 1700 cm^−1^ [44,45]. The FTIR curve of silica nanoparticles exhibits a peak at 1049 cm^−1^, which is ascribed to the Si-O–Si bond from the silicon dioxide inorganic network, with no OH-specific peaks in the 3300 cm^−1^ area. 

In the case of the produced nanocomposites, we discovered that the aforementioned peaks were slightly changed and diminished as the silica content in the polysaccharide matrix increased. The peak assigned to Si–O at 1049 cm^−1^ overlaps with the peak attributed to C–OH from salecan at 1013 cm^−1^. The resulting peaks of nanocomposites are more of a combination of the two peaks with a gradual shift to higher wavenumbers (1023 cm^−1^). Unlike other investigations which used functionalized silica nanoparticles [17,39], the present study used amorphous SiO_2_ nanoparticles which are only physically confined in biopolymeric matrices and do not participate in the crosslinking processes.

### 2.3. Swelling Behavior

**Figure 3:**
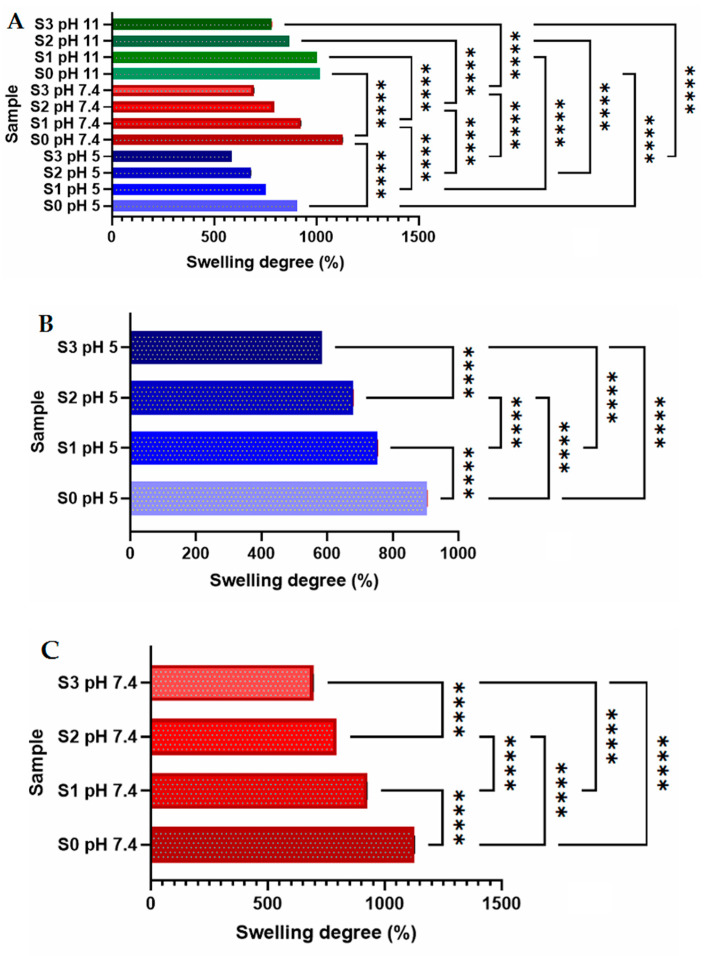

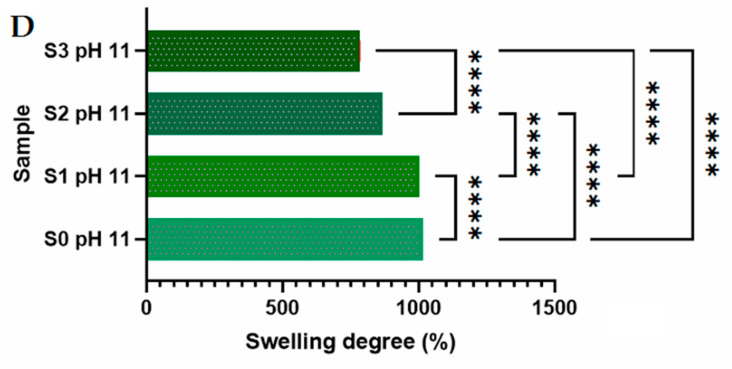
(**A**) Equilibrium swelling behavior in different pH media of the 3D printed samples. Statistical analysis (**A**): The influence of different pH media over the samples swelling degree; (**B**–**D**): The influence of composition for the samples kept in the same pH media ((**B**)-pH 5, (**C**)-pH 7.4, (**D**)-pH 11: The resulted values consist of average values with additional standard errors. Statistical significance: **** *p* < 0.0001. ONE WAY ANOVA TEST.

The hydrogel’s ability to swell in contact with fluids is a crucial characteristic for scaffolds used in tissue engineering applications since it has a significant impact on the regeneration of the new tissue and can present an important influence on the mechanical properties and the degradation rate [14,46]. The water uptake of the new 3D-printed composite hydrogels was thoroughly assessed for that aim. The swelling behavior for the studied samples was evaluated for 48 h using a simulated fluid with pH 5.5, 7.4, and 11. Figure 3 presents the values of swelling degree (SD) and illustrates how salecan and SiO_2_ content affect the water absorption capacity. The maximum amount of water was absorbed by the S0 sample (sample without silica nanoparticles) which presented the best hydrophilicity at pH 7.4.

The inclusion of silica nanoparticles in the polymeric matrix leads to the limitation of the hydrogel swelling behavior in different pH media as a consequence of the replacement of biopolymer with silica nanoparticles. Moreover, with the exception of S0, whose SD value is maximum at pH 7.4, the swelling degree was higher with the increase of the pH for all the samples because more COO– groups are forming inside the hydrogels (from the citric acid structure) [25,47]. 

All of the 3D structures that were printed have the potential to quickly absorb fluids in various pH media in about one hour. The produced 3D printed composites consist of durable biopolymer networks, evidenced by the fact that all the swelled samples retained their stability in wet settings after 24 h, the crosslinking procedure being successful in the presence of the nanofiller. Moreover, after one month, minimal degradation of less than 3% was found for samples preserved in acidic media, of ~10% for neutral pH, while the degradation reached its peak for samples preserved in the basic medium after only 30 h, regardless of sample type. This behavior is governed by the polysaccharide matrix, with a distinct behavior observed in cases where salecan crosslinked with citric acid was obtained at various concentrations of reactants [25]. 

Because hydrogel behavior is pH-dependent, this novel family of materials can therefore be used in any application that requires higher stability in an acidic environment and gradual disintegration over time in environments with greater pH’s. 

### 2.4. Thermal Properties

**Figure 4:**
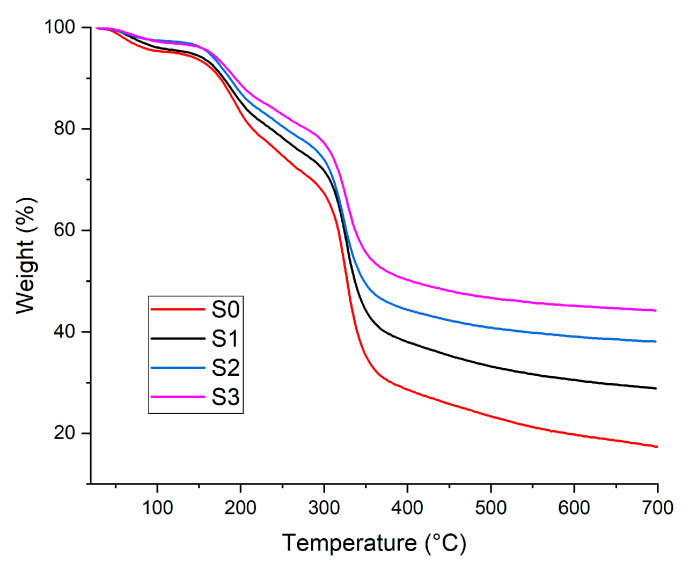
Thermal properties of the obtained sample, TGA.

To confirm that the inclusion of the silica nanoparticles can improve the thermal stability of new 3D printed composites, further TGA investigation was performed. Figure 4 presents the TGA curves of new 3D printed composites tested in nitrogen conditions with a heating rate of 10 °C/min.

Thermogravimetric parameters of the analyzed samples are presented in Table 1.

All the samples exhibited three different steps of decomposition. The first step ranging between 80 to 200 °C with a weight loss of around 10–17% was attributed to the removal of adsorbed water and slightly volatile substances [48]. The second step ranged in temperature from 200 to 320 °C and presented a weight loss of about 19–58% representing the cleavage of side chains [30]. The third decomposition stage correlated with dehydration of carbohydrate chains, which was presented between 320 and 700 °C, respectively, showed a weight loss between 26–40% [17,30]. As can be observed in Table 2, the weight loss for all the steps decreases with the addition of the silica nanoparticles, so, the sample with the highest residual mass was S3. 

### 2.5. Mechanical Properties of the Crosslinked Materials in Wet Conditions

**Figure 5:**
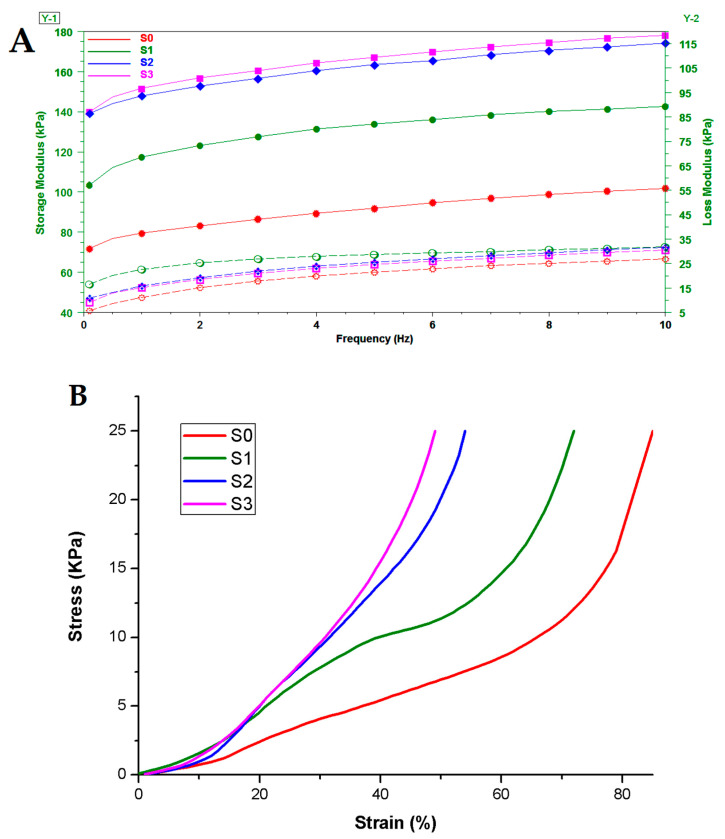
DMA analyses of the obtained hydrogels swelled at equilibrium; (**A**). Frequency sweep tests (filled symbols represent G′ and open symbols represent G″); (**B**). Stress-strain analyses.

The mechanical properties can be observed in Figure 5.

The sweeps were carried out to determine the storage modulus and loss modulus. It was observed that the storage modulus (G′) of all samples was greater than the loss modulus (G″), indicating a crosslinked state for all of the samples analyzed. With a frequency range of 0.1 to 10 Hz, the change in G′ was practically constant and still greater than that of G″, indicating that hydrogels have elastic solid characteristics and excellent stability. Generally, the storage modulus increased as SiO_2_ loadings increased. This suggested that the inclusion of silica nanofiller increased the stiffness of the salecan matrix and effectively boosted the elastic characteristics of the hydrogel nanocomposites due to the rigid inorganic nanoparticles impeding the mobility and deformability of the biopolymeric chains. This phenomenon was observed in other research studies that investigated the influence of silica nanoparticles on the mechanical properties of hydrogels [49,50,51].

A stress-strain test is an effective instrument for measuring how material changes in response to different loading situations. Stresses of ~26 KPa (according to a static force of ~6.5 N) have been applied for all samples, thus, the obtained hydrogels may satisfy the requirements of soft tissues [52,53]. The swollen cylindrical hydrogels exhibited a linear increase of stress reaching ~40–85% strain till achieving a constant compression. The crosslinked hydrogels with silica loading had a stiffer behavior that was better able to withstand stress till maximum compression. The addition of high loads of silica nanoparticles resulted in a considerable improvement in the compressive strength of the composite wet samples compared to the salecan neat sample. The origin of this phenomenon could be the decreased mobility of the biopolymer macromolecules in the presence of the inorganic filler. Another probable reason is that the absorption capacity of crosslinked hydrogels changes depending on the sample composition, with the presence of additional water molecules in the biopolymeric network impairing mechanical behavior. As depicted in Figure 5, the pure salecan sample retained more water, while the samples loaded with silica nanofiller had a lower swelling degree. 

It is important to note that the samples recovered quickly following the imposed mechanical stress showing an elastic behavior, the water ejected under stress circumstances being quickly reabsorbed. The elastic modulus of hydrogels was determined using stress-strain curves, and the three most linear stress-strain regimes were considered [54,55]. The results are presented in Table 2. 

The elastic modulus of the produced hydrogels ranged between 6.5 and 32 KPa, revealing their elastic property suited for soft tissue engineering [55]. The elastic modulus rose in the presence of silica nanoparticles across the ranges investigated, showing an increase in the stiffness of the nanocomposites. Moreover, the elastic modulus generally increased as the concentration of inorganic filler increased, with higher stress being more pronounced. The fact that the first interval deviates from the norm is intriguing, and this is very probably related to the distribution of silica particles within the hydrogel sample. Under great stress, the increased amount of silica nanoparticles are likely to restrict the movement of the biopolymer networks, resulting in the expected reliance on the amount of silica added to the system, which is consistent with other studies [56,57,58].

Thus, the inclusion of silicon dioxide nanoparticles into salecan crosslinked hydrogels can improve the mechanical behavior of the resulting hydrogel nanocomposites due to possible physical interaction between inorganic nanoparticles and salecan biopolymer chains. The salecan hydrogel’s mechanical stability in wet conditions can therefore be easily modified by adding silica nanoparticles to suit different applications such as soft tissue engineering or wound dressings.

### 2.6. Morphological Evaluation

**Figure 6:**
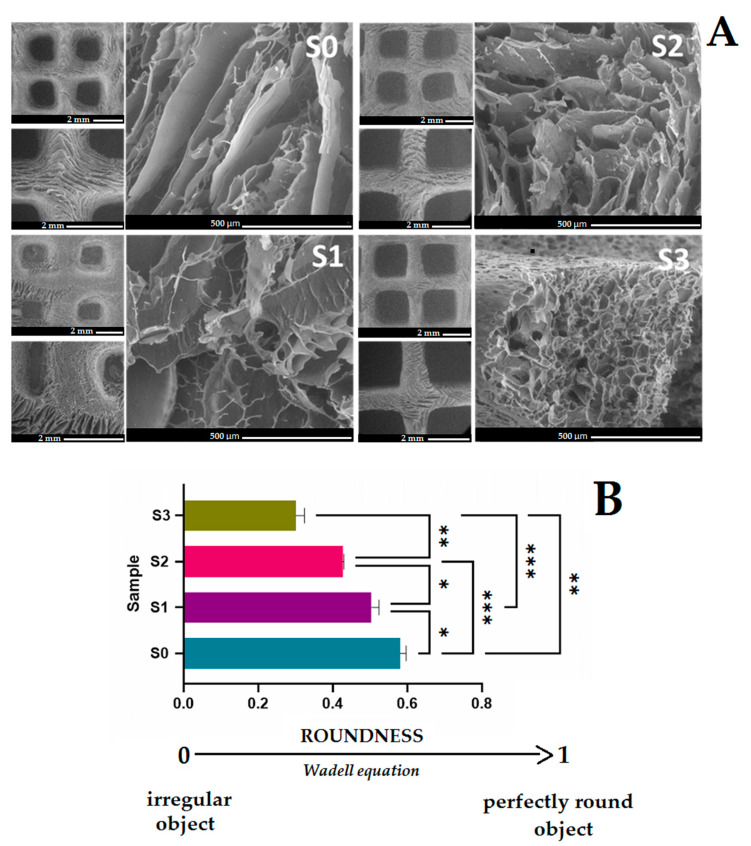
(**A**). SEM images showing the microstructure aspect of 3D printed scaffolds (30×, 50×, 250×); (**B**). The roundness of open pores is calculated using the Wadell equation. The resulting values consist of average values with additional standard errors. Statistical significance: * *p* < 0.05; ** *p* < 0.005; *** *p* < 0.0005. ONE WAY ANOVA TEST.

SEM analysis was used to examine the hydrogel’s porous microarchitecture. In Figure 6 the microstructure aspect of new 3D printed scaffolds can be observed.

The SEM images of the novel 3D printed composite reveal porous scaffolds, with an architecture strongly dependent on the composition of the hydrogels as visible in Figure 6. Furthermore, all the samples presented interconnected pores without any phase separation between the polymer matrix and silica nanoparticles. The neat salecan sample presented the spongiest structure with larger pores than salecan/SiO_2_ composite materials. The addition of silica nanoparticles to the polymer matrix causes shrinkage of the pores, creating a structure with more pores of smaller sizes. The network structure of the pores is crucial when aiming for regenerative medicine applications since it helps to guide and promote the creation of new tissue [59]. The same effect was obtained by Hu et al. [17] in their research with an increase in Fe_3_O_4_@SiO_2_ content; the average pore size was diminished. It is possible that the hydroxyl groups on nanoparticle surfaces can establish hydrogen bonds with the carboxyl and hydroxyl groups from the biopolymer/polymer chains, leading to the development of more crosslinking points as nanoparticle content increased, with a reduction in the average pore size [17,60]. Furthermore, the differences presented in SEM images suggest that in the presents of a higher content of silica nanoparticles, the 3D printed composites maintained their profile better. The roundness of open pores could be used to quantify the printing accuracy of 3D forms. Using SEM images and the H. Wadell equation, the average roundness of the open pores of the scaffolds was estimated [61]. R = 1 is obtained for a perfectly round object, but irregular shapes have values less than 1. Thus, when the macropores of 3D printed form approach a rectangular shape, R values tend to 0 while R values approaching 1 indicate that the macropores are nearly circular. The computed value of the S0 sample was ~0.58, whilst the nanocomposite samples loaded with silica nanoparticles roundness value ranged between 0.5–0.3 (Figure 6B). These differences imply that in the presence of silica nanoparticles, 3D-printed objects kept their shape better when printed. S3 had the lowest R values, indicating that the macropores are close to a rectangular shape, with the 3D printed shape matching the designed 3D model the best. This fact reinforces the observations made throughout the 3D-printing process, as well as the evaluation of the stability of the printed constructions produced when nanoparticles were used. These observations correlated well with the rheological behavior of nanocomposite inks, which showed that the silica-salecan composites’ viscosity slightly increased commensurate with their silica content.

**Figure 7:**
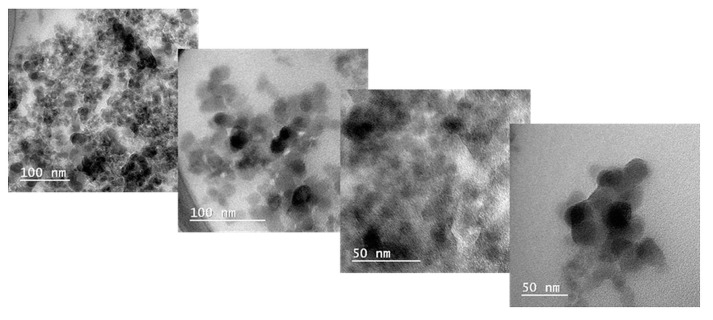
TEM images of the nanocomposite sample (S3).

Figure 7 presents the TEM images of the nanocomposite sample S3, which have a higher concentration of silica nanoparticles. From the TEM images for sample S3, nanometric agglomerates can be observed due to the presence of spherical silica particles. 

Furthermore, the TEM images revealed that the silica nanoparticles used for the development of the new 3D printed composites presented nanometer dimensions with an apparent average particle size between 10–30 nm. 

### 2.7. Antimicrobial Activity

**Figure 8:**
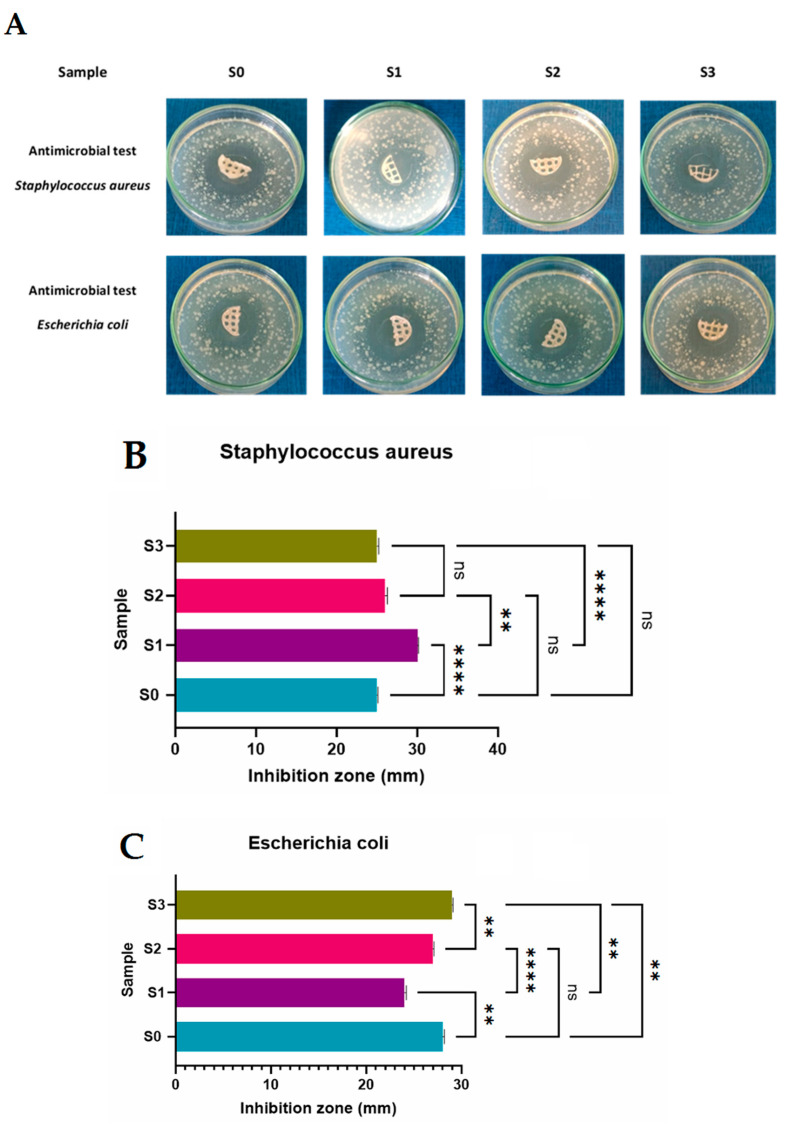
Antimicrobial activity (*Staphylococcus aureus* and *Escherichia coli*) of the 3D printed samples. (**A**). Images with the seeded samples; (**B**). Inhibition zone diameters for the samples seeded with *Staphylococcus aureus*; (**C**). Inhibition zone diameters for the samples seeded with *Escherichia coli*. Statistical significance: ns *p* < 0.5; ** *p* < 0.005, **** *p* < 0.0001. ONE WAY ANOVA TEST.

A key characteristic of many hydrogels utilized as scaffolds for regenerative medicine is their antibacterial activity [62]. All 3D printed scaffolds were tested against two gram-positive bacteria, Staphylococcus aureus and Escherichia coli (gram-negative bacteria). The antibacterial activity of every one of the obtained 3D printed salecan-based hydrogels was assessed (Figure 8).

It can be observed from Figure 8 that every sample showed antibacterial activity against the two strains that were put to the test. Due to the samples’ citric acid content, which is known to have an antibacterial effect, *Staphylococcus aureus* and *Escherichia coli* were inhibited in a detectable zone [62,63]. According to their chemical makeup, citric acid-derived polymers were shown to suppress bacterial proliferation to various degrees. Because of the leakage of unreacted citric acid molecules present in the tested samples, citrate-based polymers’ inherent antibacterial characteristics allow them to impede bacterial development [64,65,66].

All of the samples had bacterial inhibition diameters for any of the microorganisms examined, according to the results presented in Figure 8.

Thus, all the samples presented good antimicrobial activity against *Staphylococcus aureus* and *Escherichia coli* and the presence of silica nanoparticles does not alter the antimicrobial behavior of the green crosslinked polysaccharide.

### 2.8. Biological Assessment of the Crosslinked 3D Printed Polysaccharide Constructs

One of the most fundamental things to take into account when choosing materials for biomedical purposes is cytotoxicity. The influence of salecan-based hydrogels on cells’ viability was established by in vitro methods ((Live/Dead, MTT (3-[4,5-dimethylthiazol-2-yl]-2,5 diphenyl tetrazolium bromide) and LDH (lactate dehydrogenase) tests) assessed on HeLa cells. 

**Figure 9:**
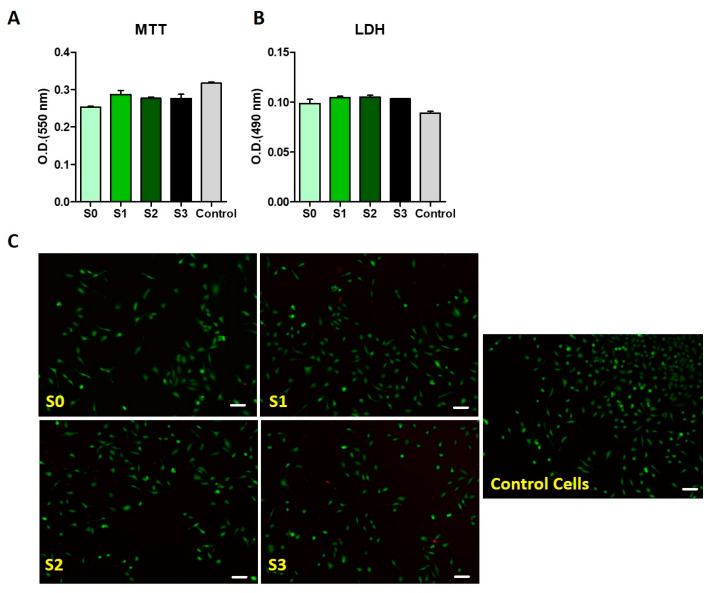
Biological assessment of the 3D printed constructs. Results were obtained from: (**A**). MTT assay, (**B**). LDH tests and (**C**). Live/Dead tests (scale bar-200 µm).

Using Live/Dead staining, the cell viability of HeLa cells, cultivated in the salecan-based hydrogels, was evaluated. After 24 h of incubation, the green fluorescence, presented in Figure 9, showed that the majority of the cells embedded in the polysaccharide hydrogels were still alive. Furthermore, the MTT test confirmed the good biocompatibility of the new 3D printed polysaccharide constructs. Additionally, no significant difference in the cytotoxicity results between the salecan hydrogels and the negative control sample was observed from the LDH test, demonstrating the non-toxicity of these 3D printed hydrogels to HeLa cells. 

The results of the MTT, LDH, and Live/Dead tests showed that the salecan-based hydrogels had great biocompatibility and can be used for biomedical applications. The outcomes are in line with those of prior research in which salecan-containing hydrogels showed promising biological characteristics [31,67,68].

## 3. Conclusions

The synthesis of nanocomposite hydrogels with appropriate rheology for the production of precise-shaped 3D constructions was accomplished. Green-crosslinked biopolymer nanosilica materials were created by adding varying quantities of silica nanoparticles to a polysaccharide hydrogel. 

According to our findings, the hydrogel’s pore size, swelling, and mechanical properties under wet circumstances can all be easily modified by varying the quantity of silica nanoparticles in the composition used for hydrogel 3D printing. Moreover, increased printing ink viscosity was obtained by adding silica nanoparticles to the salecan biopolymer matrix, which led to the creation of 3D constructions with improved shape fidelity. MTT, LDH, and Live/Dead test outcomes demonstrated the salecan-based hydrogels’ good biocompatibility and potential for usage in biomedical applications.

Due to the adaptability of the internal and external architecture, unique multi-functional hydrogels containing bioactive ingredients may be developed with particular demands and a regulated degradation profile that corresponds to the targeted tissue functionality. These investigations are presently ongoing.

Therefore, our hydrogel might be used as an easy-to-use and adaptable hydrogel for the creation of customized 3D printed constructs with a range of uses.

## 4. Materials and Methods

### 4.1. Materials and Synthesis 

Suzhou Chemicals (Suzhou, China) provided the microbial polysaccharide known as Salecan (>90% purity), and SC Remed Prodimpex SRL (Pantelimon, Romania) provided the citric acid (>99.5% purity). Sigma-Aldrich (Steinheim, Germany) supplied the silica nanoparticles (amorphous silicon dioxide, 99.8%, 12 nm average size). Using HCl 37% and NaOH (>99.3% purity) from SC Chimreactiv SRL, Romania, we created simulated biological fluids with various pH values (5.5, 7.4, and 11) in our laboratory and Na_2_HPO_4_ (>99.9% purity) from Reactivul, Voinesti, Romania. 

The new formulations were prepared according to the composition from Table 3 using an adapted preparation process [62]. 

**Figure 10:**
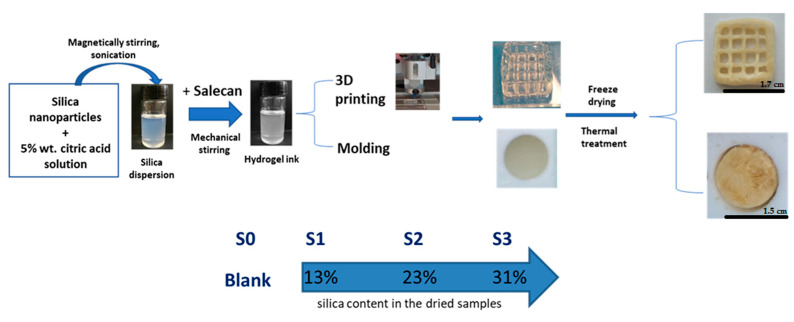
Schematic representation of the formation and characterization of the new polysaccharide-based nanocomposites.

Briefly, in order to obtain the new nanocomposite based on salecan, different concentrations of silica nanoparticles were dispersed in citric acid solution using magnetic stirring (600 rpm, 90 min, 22–25 °C using Arex heating magnetic stirrer, Velp Scientifica, Usmate Velate, Italy) and sonication treatment (5 min, 20% amplitude, 20 khz frequency, ice bath, using High Intensity Ultrasonic Processor, CPX 750, CV33, COLE PARMER, Cambridgeshire, UK); salecan was then added in the silica dispersion under mechanical stirring thus obtaining the hydrogel ink. A part of the composition was used for molding and the other part was used for 3D printing. All obtained samples were freeze-dried and then exposed to thermal treatment. The schematic synthesis steps can be observed in Figure 10. 

Four inks were developed in order to construct porous biocompatible scaffolds that could support cell attachment and proliferation because the main objective of this research study was to create a new nanocomposite printing ink that could be used in tissue engineering. A 3D bioprinter from RegenHU Ltd., Villaz-St-Pierre, Switzerland, was used to print the nanocomposite ink samples. Room temperature (25–30 °C) was used for the printing procedure. The experiments were conducted using a direct dispensing printhead and a 5 mL syringe with an attached cylindrical nozzle that has a 0.41 mm diameter at various pressures between 190 and 350 kPa. Up to 10 layers of each mixture were printed. The 3D printed hydrogel structures underwent the previously indicated processes of lyophilization and heat treatment after the additive manufacturing process. The constructions were washed to remove all of the unreacted polysaccharides, and a previously known technique was used to quantify the degree of crosslinking between the polysaccharide and citric acid [8,25,27]. The crosslinking percentage was found to be ~95% for all samples regardless of composition. This demonstrated that the biopolymer crosslinking process was not impacted by the presence of silica nanoparticles, a highly crosslinked structure being synthesized.

### 4.2. Methods

#### 4.2.1. Rheological Behavior

The shear viscosity vs. shear rate behavior was estimated using a Kinexus Pro Rheometer (Malvern, UK). The temperature of 25 °C was controlled with a Julabo CF41 cryo-compact circulator. In parallel plate geometry, the samples were put (10 mm diameter) with a gap of 0.5 mm. The shear viscosity was measured at an applied shear rate between 0.001 and 1000 s^−1^. The results were represented logarithmically.

#### 4.2.2. Fourier Transform Infrared Spectrometry (FT-IR)

The samples were structurally evaluated using Vertex 70 Bruker FTIR spectrometer (Billerica, MA, USA). All the FT-IR analyses were achieved in the 4000–600 cm^−1^ wavelength scale. The grounded samples were used for qualitative FTIR analysis in ATR mode.

#### 4.2.3. Swelling Behavior 

The swelling degree of the obtained 3D printed composites was analyzed by incubation in simulated biological fluids having different pH values (pH = 5.5; 7.4; 11) and a temperature of 37 °C. After a predicted time, the samples were removed from fluids, and filter paper was used to remove any surface-adsorbed water. The increase in weight (w − w0) from the initial weight (w0) represented the degree of swelling. 

#### 4.2.4. Thermo Gravimetric Analysis (TGA)

With a NETZSCH TG 209 F1 Libra instrument (Selb, Germany) (controlled atmosphere utilizing a nitrogen flow rate of around 20 mL/min, scanning from 25 to 700 °C and a heating degree of 10 °C/min), the thermogravimetric analysis of 3D printed nanocomposite was evaluated in triplicate. The samples utilized for all measurements ranged in mass from 3.5 to 5 mg.

#### 4.2.5. Scanning Electron Microscopy (SEM)

The internal structure and morphology of the 3D printed nanocomposites were examined using the scanning electron microscope ESEM-FEI Quanta 200 (Eindhoven, The Netherlands). All the scaffolds were studied without any coating. 

Wadell’s Equation (1) was used to calculate the roundness of open pores (R). R is therefore equal to the average radius of curvature of the four corners of the 3D construct open pores to the radius of the greatest inscribed circle of the analyzed pore, as specified in Equation (1):

(1) $$R=\left(\frac{1}{n}\sum_{i=1}^{n}ri\right)/rmax$$

where *ri* = radius of the corner curvature, *rmax* = radius of the greatest inscribed circle, *n*—number of corners (*n* = 4). The R-value for every 3D construct is the estimated average of the open pores shown in the SEM picture. R is greater than 0 for all other objects and equals 1 for perfectly spherical objects [61,69].

#### 4.2.6. Transmission Electron Microscopy (TEM)

The freeze-dried 3D printed composites were examined using transmission electron microscopy (TEM). On a TECNAI F20 G2 TWIN Cryo-TEM (FEI, Hillsboro, OR, USA), all of the samples were examined in BF-TEM (Bright Field Transmission Electron Microscopy) mode at an accelerating voltage of 200 kV.

#### 4.2.7. Mechanical Tests 

The resulting hydrogel samples’ mechanical behavior was assessed using a DMA Q800 TA Instruments (New Castle, DE, USA). The analyses were conducted at 37 °C in compression mode using cylindrical samples with a 15 mm diameter and ~8 mm thickness. All of the equilibrium swollen composites were compressed, with a ramp force of 0.2 N/min to 6.5 N/min till a constant strain plateau was registered. 

Elastic modulus was calculated from stress-strain curves using the following equation E = σ/ε, where compressive stress is denoted as σ (N/m^2^) and ε represents the related strain. Three linear regions of stress-strain curves were considered to calculate the elastic modulus, namely: 1–6, 20–25, and 25–35% strain compressions.

Dynamic frequency sweeps were carried out over a frequency range of 0.1–10 Hz with a continuous strain of 0.1% (in the linear viscoelastic area) at 25 °C in order to record the storage (G′) and loss (G″) moduli of the water-swollen samples at equilibrium.

#### 4.2.8. Antimicrobial Activity

Two standard strains from the ICPI Institute’s Microbiology Department collection—*Escherichia coli* ATCC 11229, a Gram-negative bacteria strain, and *Staphylococcus aureus* ATCC 25923, a Gram-positive bacterium strain—were used to determine the antibacterial activity for the 3D printed nanocomposites. Using a modified spot diffusion approach, the antibacterial properties were qualitatively screened. Our prior research study included a full description of this method’s procedure [70]. Following 24–48 h of microbial cultures developed on Muller Hinton agar (MHA), bacterial and yeast suspensions of (1–5) × 10^8^ µcf/mL (corresponding with 0.5 McFarland standard density) were obtained. These suspensions were put in Petri dishes, left at room temperature to ensure equal diffusion of the compound in the medium, and then incubated with 3D printed composites at 37 °C for 24–48 h. The samples’ inhibitory zone diameters were evaluated in triplicate after 24 h.

#### 4.2.9. Biological Assessment of the 3D Printed Salecan-Based Hydrogels

The biocompatibility of the materials was tested on HeLa cells. Cells were cultivated at a density of 10^5^ cells/ well in Dulbecco’s Modified Eagle Medium with 10% fetal bovine serum. The materials were UV sterilized and added on top of the cells for 24 h. Cytotoxicity was measured using the LDH Cytotoxicity kit (Sigma) following the manufacturer’s instructions. Absorbance was read at λ = 490 nm using a NanoQuant Infinite M200 Pro instrument. The viability of the cells was analyzed using the Live/Dead assay (cat. No. L3224). Imaging was performed at λ = 494/517 (live cells) and at λ = 517/617 (dead cells) using a Zeiss fluorescence microscope.

Cell proliferation was quantified using the CyQUANT™ MTT Cell Viability Assay (Thermos Scientific, Zeiss Oberkochen, Baden-Wurttenberg, Germany) following the manufacturer’s instructions.

#### 4.2.10. Statistical Analyses

The data are presented as mean standard deviation. A one-way ANOVA was used to assess the significance of differences. If the *p*-value was less than 0.05, the significance was evaluated.

## Figures and Tables

**Table 1:** Thermogravimetric of the samples.

Samples	Step 1		Step 2		Step 3	
	T, °C	Weight Loss, %	T, °C	Weight Loss, %	T, °C	Weight Loss, %
S0	80–200	16.81	200–320	57.49	320–700	39.86
S1		11.95		22.07		34.35
S2		10.84		22.31		26.72
S3		9.29		18.48		26.10

**Table 2:** Elastic modulus calculated from stress-strain curves.

Sample			Elastic Modulus (KPa)						
	1–6 KPa	STDV	R^2^	20–25 KPa	STDV	R^2^	25–35 KPa	STDV	R^2^
S0	6.57	±0.66	0.9906	12.74	±0.44	0.9990	13.46	±0.10	0.9971
S1	15.35	±2.10	0.9979	24.37	±0.98	0.9988	25.90	±0.18	0.9977
S2	7.32	±1.29	0.9913	27.38	±1.45	0.9998	31.09	±1.36	0.9993
S3	9.42	±2.36	0.9988	27.47	±1.50	0.9977	32.14	±1.88	0.9984

**Table 3:** Composition of the obtained composites.

Sample	Salecan [g]	Citric Acid Solution 5% [mL]	Silica Nanoparticles [g]
S0	1.125	15	-
S1	1.125	15	0.281
S2	1.125	15	0.562
S3	1.125	15	0.843

## Data Availability

Not applicable.
